# A process parameters dataset for the extrusion 3D printing of nutraceutical oral dosage forms formulated with monoglycerides oleogels and phytosterols mixtures

**DOI:** 10.1016/j.dib.2019.104805

**Published:** 2019-11-16

**Authors:** Ivana M. Cotabarren, Sofia Cruces, Camila A. Palla

**Affiliations:** a Departamento de Ingeniería Química (DIQ) - Universidad Nacional del Sur (UNS), Bahía Blanca, Buenos Aires, Argentina; b Planta Piloto de Ingeniería Química (PLAPIQUI, UNS-CONICET), Bahía Blanca, Buenos Aires, Argentina

**Keywords:** Printing settings, 3D printing, Nutraceuticals, Oleogels, Phytosterols

## Abstract

We report the parameter settings used in different 3D printing tests carried out to evaluate the production of nutraceutical oral forms by using mixtures of monoglycerides oleogels and phytosterols as printing materials. The printer employed was an ad-hoc extrusion 3D printing system adapted from a Prusa printer. The dataset here informed would serve as a starting point for the implementation of the 3D printing process to fabricate products using oleogels or printing materials with similar characteristics. This data is related to our recent research article entitled “Extrusion 3D printing of nutraceutical oral dosage forms formulated with monoglycerides oleogels and phytosterols mixtures” [1].

**Table:** Specifications Table

Subject	Process Chemistry and Technology
Specific subject area	3D printing of nutraceuticals
Type of data	Table Image
How data were acquired	Printing was performed in an ad-hoc extrusion 3D printer adapted from a Prusa with Repetier-Host (V2.0.5) [2] software for parameter settings. Image acquisition by digital camera. Temperatures were recorded using a digital thermometer.
Data format	Raw
Parameters for data collection	Normal printing conditions
Description of data collection	Through the printer software (Repetier Host V2.0.5, [2]) the following variables were registered: Syringe temperature set-point, first layer extrusion width, filament diameter, in fill percentage, shell thickness, flow percentage, z-hop, layer thickness, first layer thickness, printing speed, infill pattern, and infill overlap. It was also registered if the cooling fan was on, the use of Peltier as a refrigerating system, the type of nozzle used, ambient temperate, build platform temperature, nozzle tip temperature, pictures of failed printing tests and printed forms, and mass of printed forms.
Data source location	Institution: Planta Piloto de Ingeniería Química (PLAPIQUI, UNS-CONICET) City/Town/Region: Bahía Blanca, Buenos Aires Country: Argentina
Data accessibility	With the article
Related research article	Cotabarren, I.M., Cruces, S., Palla, C.A., 2019. Extrusion 3D printing of nutraceutical oral dosage forms formulated with monoglycerides oleogels and phytosterols mixtures. Food Res. Int. https://doi.org/10.1016/j.foodres.2019.108676

**Table:** 

**Value of the Data** • This article provides data for the first time for the setting conditions required in an extrusion 3D printer system to successfully print oral forms in which the printable material consists of molten mixtures of monoglycerides oleogels and phytosterols. • The provided information shows the relationship between some printing parameters, the gel point of printing materials, and the printability under these setting conditions. • This dataset could be used to assist extrusion 3D printer users in the setting of printing parameters, which need to be adapted to the specific material properties of the particular process. • These data can be used to explore different printing settings that allow to optimize the process of obtaining nutraceutical oral forms by using monoglycerides oleogels and higher phytosterols ratio or oleogels incorporating other different liposoluble molecules. • The dataset here informed would serve as a starting point for the implementation of the 3D printing process to fabricate products using oleogels or printing materials with similar characteristics. • These data confirm the feasibility of applying 3D printing technologies focused on food and nutraceutical products, which is a very recent field of investigation.

## 1 Data

The complete set of printing parameters for the extrusion 3D printing of nutraceutical oral forms formulated with different mixtures of monoglycerides oleogels and phytosterols are shown in Table 1. The information includes:

• *Mixture*: 8 different mixtures formulated using 10 or 20 %wt of monoglycerides in high oleic sunflower oil (oleogel) and ratios between 20 and 50 %wt of phytosterols/oleogel were tested. The mixtures were coded according to the monoglycerides concentration and the phytosterols/oleogel ratio (e.g., M10-20 contains 10 %wt of monoglycerides and 20 %wt of phytosterols/oleogel).
• *Syringe temperature set-point* ($T_{set}$).
• *Measured syringe temperature* ($T_{s}$).
• *Measured nozzle temperature* ($T_{n}$).
• *Ambient temperature* ($T_{amb}$).
• *Build platform temperature* ($T_{P}$).
• *Mass* of the successful printed oral dosage forms.
• *First layer extrusion width*: it establishes the width of the line of deposited material as a percentage (100% default setting).
• *Filament diameter*: in a commercial Prusa it is the diameter of the used polymer filament. Since this is an ad-hoc extrusion printer, this value was set according to the amount of material that is pushed out of the syringe in each motor step.
• *Infill density percentage*: this parameter states how much material is to be printed in the inner lines of each layer; the higher the infill density is, the more lines the extruder will print in each layer.
• *Shell thickness*: it is the width of the outer perimeter printed in each layer.
• *Flow percentage*: refers to how much material is to be extruded during the printing process.
• *Z-hop*: this is the vertical distance that the nozzle retracts when moving without extruding material. It prevents or reduces the scratching of the object surface.
• *Layer thickness*: defines the height of each layer of the deposited material.
• *First layer thickness*: defines the height of the first layer of the deposited material.
• *Printing speed*: it is the nozzle displacement velocity.
• *Infill pattern*: refers to the movements described by the nozzle when printing the interior paths of each layer. It can take two settings: linear, which means that the interior paths are longitudinal lines starting at one side of the object; or concentric, in which the nozzle describes concentric lines starting from the outer perimeter of the object.
• *Infill overlap percentage*: it is the overlap between the consecutive infill lines expressed as percentage.
• *Fan speed*: it represents the cooling fan operation. It was either set OFF or ON (for a certain time) during the printing process. For example, “ON 30% of printing” indicates that fan was running during 30% of the time the printing last. In all the specified cases, the fan was set ON during the last part of the process.
• *Type of nozzle*: 3 different nozzles were tested, each one presenting different dimensions (see Cotabarren et al. [1] for more detail).
• *Peltier* use: it indicates either if the build platform was refrigerated or not by a Peltier system.
• *Picture*: images for all the successful printed oral forms are included. To further illustrate the printing process, several images for the non-successful printed oral forms are also included.

**Table 1:** Process parameters settings and oral dosage pictures for de extrusion 3D printing of mixtures containing monoglycerides oleogels and phytosterols.

Mixture	$T_{\mathrm{set}}$(°C)	$T_{n}$(°C)	$T_{s}$(°C)	$T_{\mathrm{amb}}$(°C)	$T_{P}$(°C)	Mass (g)	1° layer Extrusion Width	Filament Diameter (mm)	Infill Density (%)	Shell Thickness (mm)	Flow (%)	Z-hop (mm)	Layer Thickness (mm)	1° layer Thickness (mm)	Printing Speed (mm/s)	Infill Pattern	Infill Overlap (%)	Fan Speed	Nozzle Type	Peltier	Picture
M20-50	85	62	80	24	n/m	n/m	100	18.5	100	0.8	100	0	0.2	0.3	5	linear	1	OFF	A	NO	n/m
M10-20	95	68	92	25	n/m	n/m	100	18.5	100	0.8	100	0	0.2	0.3	5	linear	1	OFF	A	NO	n/m
M10-20	100	70	82	25	n/m	n/m	100	18.5	100	0.8	100	0	0.2	0.3	5	linear	1	OFF	A	NO	
M10-20	115	80	96	26	n/m	n/m	100	18.5	100	0.8	100	0	0.2	0.3	5	linear	1	ON	B	NO	
M10-20	115	78	85	26	n/m	n/m	100	18.5	100	0.8	100	0	0.2	0.3	5	linear	1	OFF	B	NO	n/m
M10-20	105	68	88	20	n/m	n/m	100	18.5	100	0.8	100	0.2	0.2	0.3	5	linear	1	OFF	C	NO	n/m
M10-20	105	66	88	20	n/m	n/m	100	17.5	100	0.8	100	1	0.3	0.3	5	linear	1	OFF	C	NO	
M10-20	105	78	92	23	n/m	n/m	100	17	100	0.8	110	1	0.3	0.3	5	linear	1	OFF	C	NO	
M10-20	110	55	90	24	n/m	n/m	100	16.5	100	0.8	110	1	0.3	0.3	5	linear	1	OFF	C	NO	
M10-20	110	56	85	22	n/m	n/m	100	9	47	0.8	110	1	0.3	0.3	5	linear	1	OFF	C	NO	
M10-20	110	58	90	25	n/m	n/m	100	12	47	0.8	110	1	0.3	0.3	5	linear	1	OFF	C	NO	
M10-20	110	53	91	24	n/m	n/m	100	12	47	0.8	110	1	0.3	0.3	5	linear	1	OFF	C	NO	
M10-20	110	56	86	26	n/m	n/m	100	12	47	0.8	110	1	0.3	0.3	5	linear	1	OFF	C	NO	n/m
M10-20	110	56	86	25	n/m	n/m	100	12	47	0.8	110	1	1	0.35	5	linear	1	OFF	A	NO	
M10-20	110	56	86	24	n/m	n/m	110	14	47	0.8	100	1	1	0.2	5	linear	1	OFF	A	NO	
M10-20	110	56	86	24	n/m	n/m	90	14	47	0.8	100	1	1	0.2	5	linear	1	OFF	A	NO	
M10-20	110	56	86	24	n/m	n/m	90	15	47	0.8	100	1	1.5	0.25	5	linear	1	OFF	A	NO	
M20-20	110	73	88	21	n/m	n/m	110	14	47	0.8	100	1	1	0.2	5	linear	1	OFF	A	NO	
M20-20	110	76	97	20	n/m	n/m	110	14	47	0.8	100	1	1	0.3	5	linear	1	OFF	A	NO	
M10-50	110	76	89	21	n/m	n/m	110	14	47	0.8	100	1	0.2	0.2	5	linear	1	OFF	A	NO	
M10-50	110	74	89	20	n/m	n/m	110	14	47	0.8	100	1	1	0.2	5	linear	1	OFF	A	NO	
M10-50	115	71	88	20	n/m	n/m	110	14	47	0.8	100	1	0.2	0.3	5	linear	1	OFF	A	NO	
M10-50	115	68	90	21	n/m	n/m	110	14	47	0.8	115	1	0.2	0.3	5	linear	1	OFF	A	NO	
M10-50	115	70	88	23	n/m	n/m	110	13	47	0.8	118	1	0.2	0.3	8	linear	1	OFF	A	NO	
M10-20	110	70	90	23	n/m	n/m	110	14	47	0.8	100	1	1	0.2	5	linear	1	OFF	A	NO	n/m
M10-20	110	70	90	22	n/m	n/m	112	14	47	0.8	105	1	1	0.2	5	linear	1	OFF	A	NO	
M10-20	110	68	89	22	n/m	n/m	112	13.5	47	0.8	110	1	1	0.2	5	linear	1	OFF	A	NO	
M10-20	110	68	89	23	n/m	n/m	112	13.5	47	0.8	110	1	1	0.2	5	linear	1	OFF	A	NO	
M10-20	110	74	96	26	n/m	n/m	108	13.5	47	0.8	100	1	1	0.2	5	linear	1	OFF	A	NO	
M10-20	110	79	100	25	n/m	n/m	108	14.5	47	0.8	100	1	1	0.2	5	linear	1	OFF	A	NO	n/m
M10-20	110	81	90	26	n/m	n/m	110	14	47	0.8	100	1	1	0.2	5	linear	1	OFF	A	NO	n/m
M20-30	110	67	98	24	9	n/m	110	14	47	0.8	100	1	1	0.2	5	linear	1	OFF	A	YES	n/m
M20-30	110	67	98	24	9	n/m	110	14	47	0.3	100	1	1	0.2	5	linear	1	OFF	A	YES	
M20-30	110	65	98	24	9	1.181	110	14	47	0.8	100	1	1.2	0.2	5	linear	1	OFF	A	YES	
M20-30	110	67	98	24	9	1.164	110	14	47	0.8	100	1	1	0.2	5	linear	1	OFF	A	YES	
M20-30	110	67	100	24	9	1.130	110	14	47	0.8	100	1	1	0.2	5	concentric	1	OFF	A	YES	
M20-30	110	67	98	24	9	1.102	110	14	47	0.3	100	1	1	0.2	5	concentric	1	OFF	A	YES	
M10-20	110	67	98	23	9	1.393	110	14	47	0.3	100	1	1	0.2	6	concentric	2	OFF	A	YES	
M10-20	110	67	98	23	9	n/m	110	14	47	0.4	100	1	1	0.2	6	concentric	2	OFF	A	YES	
M10-20	110	67	98	23	9	n/m	110	14	47	0.3	100	1	1.2	0.2	6	concentric	4	OFF	A	YES	
M10-20	110	67	98	23	9	1.168	110	14	47	0.3	100	1	1	0.2	6	concentric	2	OFF	A	YES	
M10-20	110	67	98	23	9	1.165	110	14	47	0.3	100	1	1	0.2	6	concentric	2	OFF	A	YES	
M10-20	110	67	98	23	9	1.165	110	14	47	0.3	100	1	1	0.2	6	concentric	2	OFF	A	YES	
M10-20	110	67	98	23	9	1.151	110	14	47	0.3	100	1	1	0.2	6	concentric	2	OFF	A	YES	
M20-20	110	71	90	25	8	1.3294	110	14	47	0.3	100	1	1	0.3	5	concentric	2	OFF	A	YES	
M20-20	110	71	90	25	8	1.1475	110	14	47	0.3	100	1	1	0.3	5	concentric	2	OFF	A	YES	
M20-20	110	71	90	25	8	1.2502	110	14	47	0.3	100	1	1	0.3	5	concentric	2	OFF	A	YES	
M20-20	110	71	90	25	8	1.2023	110	14	47	0.3	100	1	1	0.3	5	concentric	2	OFF	A	YES	
M20-20	110	71	90	25	8	1.2629	110	14	47	0.3	100	1	1	0.3	5	concentric	2	OFF	A	YES	
M10-30	110	72	92	24	7	n/m	110	14	47	0.3	100	1	1	0.3	6	concentric	2	OFF	A	YES	
M10-30	110	72	92	24	7	n/m	110	14	47	0.3	100	1	1	0.3	5	concentric	2	OFF	A	YES	n/m
M10-30	110	72	92	24	7	n/m	110	14	47	0.3	95	1	1	0.2	5	concentric	2	OFF	A	YES	n/m
M10-30	110	72	92	24	7	n/m	110	14	47	0.3	90	1	1	0.2	5	concentric	2	OFF	A	YES	n/m
M10-30	110	72	92	24	7	n/m	110	14	47	0.3	80	1	1	0.2	5	concentric	2	OFF	A	YES	n/m
M10-30	110	72	92	24	7	n/m	110	14	47	0.3	80	1	1	0.2	5	concentric	2	OFF	A	YES	n/m
M10-30	110	72	92	24	7	n/m	110	14	47	0.3	85	1	1	0.2	5	concentric	2	OFF	A	YES	n/m
M10-30	110	72	92	24	7	n/m	110	14	23	0.25	100	1	1	0.2	5	concentric	2	OFF	A	YES	n/m
M10-30	110	72	92	24	7	n/m	110	14	30	0.3	100	1	1	0.2	5	concentric	2	OFF	A	YES	n/m
M10-30	110	72	92	24	7	1.1577	110	14	40	0.3	100	1	1	0.2	5	concentric	2	OFF	A	YES	
M10-30	110	72	92	24	7	1.1023	110	14	40	0.3	100	1	1	0.2	5	concentric	2	OFF	A	YES	
M10-30	110	72	92	24	7	n/m	110	14	38	0.3	100	1	1	0.2	5	concentric	2	OFF	A	YES	n/m
M10-30	110	72	92	24	7	0.7748	110	14	40	0.3	100	1	1	0.2	4	concentric	2	OFF	A	YES	
M10-30	110	72	92	24	7	n/m	110	14	40	0.3	100	1	1	0.2	4	concentric	2	OFF	A	YES	n/m
M10-30	110	72	92	24	7	1.0183	110	14	40	0.3	100	1	1	0.2	5	concentric	2	OFF	A	YES	
M10-30	110	72	92	24	7	1.0335	110	14	40	0.3	100	1	1	0.2	5	concentric	2	OFF	A	YES	
M10-30	110	72	92	24	7	n/m	110	14	40	0.3	100	1	1	0.2	5	concentric	2	OFF	A	YES	n/m
M20-40	110	74	95	24	5	n/m	110	14	47	0.3	100	1	1	0.2	5	concentric	2	OFF	A	YES	n/m
M20-40	110	74	95	24	5	n/m	110	14	47	0.3	100	1	1	0.2	5	concentric	2	ON	A	YES	n/m
M20-40	110	74	95	24	5	1.082	110	14	40	0.3	100	1	1	0.2	5	concentric	2	OFF	A	YES	
M20-40	115	78	100	24	5	n/m	110	14	40	0.3	100	1	1	0.2	5	concentric	2	OFF	A	YES	n/m
M20-40	115	78	100	24	5	n/m	110	14	35	0.3	100	1	1	0.2	5	concentric	2	OFF	A	YES	
M20-40	115	78	100	24	5	n/m	110	14	35	0.3	100	1	1	0.2	5	concentric	2	ON 30% of printing	A	YES	
M20-40	115	78	100	24	10	1.0595	110	14	35	0.3	100	1	1	0.2	5	concentric	2	ON 30% of printing	A	YES	
M20-40	115	78	100	24	10	1.102	110	14	35	0.3	100	1	1	0.2	5	concentric	2	ON 30% of printing	A	YES	
M10-40	115	81	99	24	7	n/m	110	14	47	0.3	100	1	1	0.2	5	concentric	2	OFF	A	YES	
M10-40	115	81	99	24	7	n/m	110	14	47	0.3	100	1	1	0.2	5	concentric	2	OFF	A	YES	
M10-40	115	81	99	24	7	n/m	110	14	40	0.3	100	1	1	0.2	5	concentric	2	OFF	A	YES	
M10-40	115	77	99	24	7	n/m	110	14	30	0.3	100	1	1	0.2	5	concentric	2	OFF	A	YES	
M10-40	118	81	104	24	7	1.0736	110	14	30	0.3	100	1	1	0.2	5	concentric	2	OFF	A	YES	
M10-40	118	81	104	24	7	1.0361	110	14	30	0.3	100	1	1	0.2	5	concentric	2	OFF	A	YES	
M10-40	118	81	104	24	7	1.0183	110	14	30	0.3	100	1	1.2	0.2	5	concentric	2	OFF	A	YES	
M20-50	115	76	98	24	5.5	n/m	110	14	35	0.3	100	1	1	0.2	5	concentric	2	OFF	A	YES	
M20-50	118	82	104	24	5.5	n/m	110	14	35	0.3	100	1	1	0.2	5	concentric	2	OFF	A	YES	
M20-50	125	82	105	24	5.5	n/m	110	14	35	0.3	100	1	1	0.2	5	concentric	2	OFF	A	YES	

n/m = not measured.

## 2 Experimental design, materials, and methods

The printing procedure used to obtain the solid forms consisted of the following steps:

1. A CAD file was generated to define the geometry of the nutraceutical oral solid form by using a free CAD software [3]. The design was performed following closely the dimensions of 1 g commercial pharmaceutical tablets.
2. The generated STL file was imported to the slicer software Repetier Host V2.0.5 [2] and all the printing parameters were set (i.e., layer thickness, material infill, material flow, printing speed, syringe temperature, build platform temperature, etc.).
3. The syringe was preheated to a set temperature ($T_{set}$), higher than the mixture gel temperature in order to maintain molten the printing material. With a manual sensor, the temperature along the heating system and the metal nozzle was registered ($T_{s}$ and $T_{n}$). For the gel point of the mixtures used as printing materials please refer to Cotabarren et al. [1].
4. The molten mixture was charged into the syringe by reverse action of the plunge motion motor, starting from the plunge in the lower position, in order to reduce the air volume to its minimum.
5. For the cases with Peltier refrigeration, the build platform temperature ($T_{p}$) was allowed to stabilize.
6. The STL file was sent to the printer and the extrusion process begun.
7. After printing, pictures of the failed printing tests or the fabricated oral forms were taken and their weight registered.
